# Electroreduction of CO to 2.8 A cm⁻^2^ C_2+_ Products: Maximizing Efficiency with Minimalist Electrode Design Featuring a Mesopore‐Rich Hydrophobic Copper Catalyst Layer

**DOI:** 10.1002/advs.202405938

**Published:** 2024-08-26

**Authors:** Silu Chen, Ben Rowley, Ramesha Ganganahalli, Boon Siang Yeo

**Affiliations:** ^1^ Department of Chemistry Faculty of Science National University of Singapore 3 Science Drive 3 Singapore 117543 Singapore; ^2^ Energy Transition Campus Amsterdam Grasweg 31, 1031 HW Amsterdam The Netherlands; ^3^ Shell India Markets Private Ltd. Plot No. 7, Bengaluru Hardware Park, Mahadeva, Kodigehalli Bangalore 562149 India

**Keywords:** CO reduction, copper, electrocatalysis, hydrophobicity, nanoparticles

## Abstract

This work shows how hydrophobicity and porosity can be incorporated into copper catalyst layers (CLs) for the efficient electroreduction of CO (CORR) in a flow cell. Oxide‐derived (OD) Cu catalysts are synthesized using K^+^ and Cs^+^ as templates, termed respectively as OD‐Cu‐K and OD‐Cu‐Cs. CLs, assembled from OD‐Cu‐K and OD‐Cu‐Cs, exhibit enhanced CORR performance compared to “unmodified” OD‐Cu CL. OD‐Cu‐Cs can notably reduce CO to C_2+_ products with Faradaic efficiencies (FE) as high as 96% (or 4% FE H_2_). During CO electrolysis at −3000 mA cm^−2^ (−0.73 V vs reversible hydrogen electrode), C_2+_ products and the alcohols are formed with respective current densities of −2804 and −1205 mA cm^−^^2^. The mesopores in the OD‐Cu‐Cs CL act as barriers against electrolyte flooding. Contact angle measurements confirm the CL's hydrophobicity ranking: OD‐Cu‐Cs > OD‐Cu‐K > OD‐Cu. The enhanced hydrophobicity of a catalyst is proposed to allow more triple‐phase (CO‐electrolyte‐catalyst) interfaces to be available for CORR. This study shows how the pore size‐hydrophobicity relationship can be harvested to guide the design of a less‐is‐more Cu electrode, which can attain high CORR current density and selectivity, without the additional use of hydrophobic polytetrafluoroethylene particles or dopants, such as Ag.

## Graphical abstract

A hydrophobic copper catalyst layer, created solely by the presence of numerous mesopores, is developed. This simple, but highly effective design slows down the penetration of the electrolyte, thus avoiding electrode flooding. Consequently, the catalyst layer can reduce CO to C_2+_ products with a partial current density (*j*_C2+_) up to −2.8 A cm^−2^.

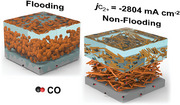

## 1 Introduction

**Scheme 1:**
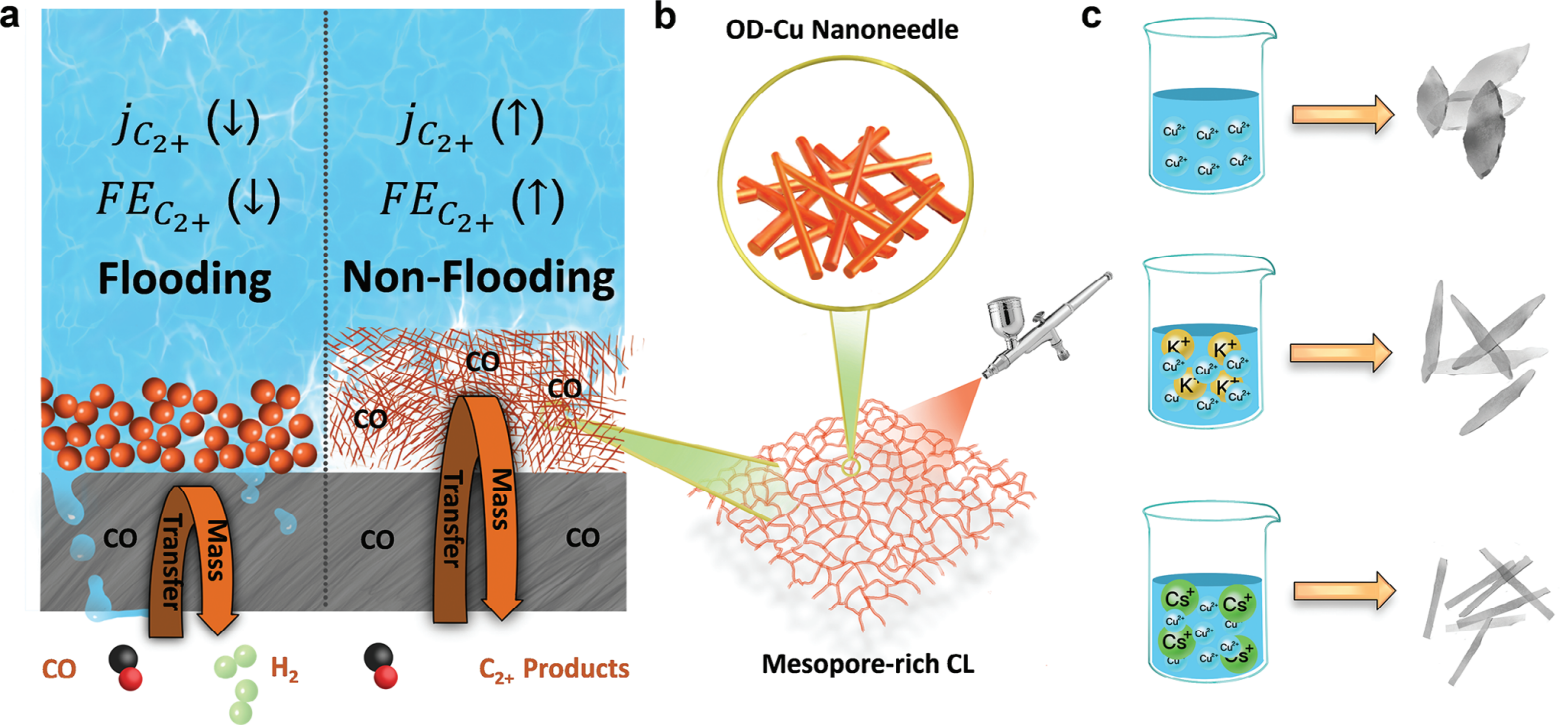
Schematic diagrams for a) flooded and nonflooded GDEs. b) Mesopore‐rich catalyst layers (CL), and c) formation of CuO, CuO‐K, and CuO‐Cs.

CO_(2)_ electroreduction (CO_(2)_RR) in flow cells, powered by renewable electricity, has the potential to sustainably produce valuable chemicals at the industrial scale. The catalyst layer (CL) is typically deposited onto gas diffusion electrodes (GDE). A significant problem that negatively affects the performance of these GDEs is electrode flooding, where the electrolyte floods the electrode surface, infiltrates the gas diffusion layer, and impedes CO transport, thereby reducing reaction efficiency (**Scheme** **1**a).^[^ ^1^, ^2^ ^]^ To address this challenge, meticulous electrode design is essential.

Current works to prevent electrode flooding during CO_(2)_RR have focused on using hydrophobic surfaces and coatings to create pathways for gas diffusion while repelling excess electrolyte. Toward this end, hydrophobic polytetrafluoroethylene (PTFE) and carbon nanoparticles have been mixed with the catalyst particles.^[^ ^3^, ^4^ ^]^ Reyes et al. changed the hydrophobicity of CO_2_RR working electrodes by adjusting the PTFE content, and showed that Ag GDEs prepared using 29% PTFE had higher CO selectivity compared with Ag GDEs with 6% PTFE.^[^ ^5^ ^]^ Xing et al. similarly designed electrodes with localized hydrophobic centers by dispersing PTFE particles within the CL, where the PTFE repels the liquid electrolyte and maintains gas pockets, thus improving the mass transport of CO_2_ and its electroreduction.^[^ ^6^ ^]^ Geng et al. used polystyrene (PS) spheres as pore formers to synthesize porous Cu_2_O spheres and found that efficient CO_2_ transportation, surface hydrophobicity and abundant triple‐phase interfaces could be achieved on them.^[^ ^7^ ^]^ Consequently, CO_2_ could be reduced to C_2+_ products with an ∼75% Faradaic efficiency (FE) during electrolysis at −1.0 A cm^–2^. Alkanethiols can also be used as a hydrophobic coating in electrode preparation. Wakerley et al. synthesized a hydrophobic surface by treating hierarchically‐structured Cu dendrites with 1‐octadecanethiol. This electrode reduced CO_2_ to ethylene and ethanol with FEs of 56% and 17%, respectively, at neutral pH, outperforming its nontreated counterpart which only achieved 9% FE ethylene and 4% FE ethanol.^[^ ^8^ ^]^ However, as dielectric materials, while PTFE, PS, and alkanethiols enhance the hydrophobicity of electrodes, they can weaken the electrode's conductivity if used excessively. These compounds can also block the active sites of the catalyst.^[^ ^9^, ^10^ ^]^ PTFE decomposition at negative potentials has been further reported.^[^ ^11^ ^]^

Copper CL has a generally hydrophobic surface,^[^ ^12^ ^]^ which can be further enhanced by incorporating small pores into its structure. Specifically, as the size of the pores decreases, the capillary pressure will increase. This will make it difficult for an electrolyte to penetrate and flood the CL.^[^ ^13^, ^14^, ^15^ ^]^ This phenomenon is quantified by the Young–Laplace equation where the critical burst‐through pressure through a pore is expressed as *P*_liquid_ −  *P*_gas_ =  4σsin (θ_a_ − 90°)/d, where σ is the surface tension of the electrolyte (74.4 mN m^−1^ for 1 m KOH), d is the pore diameter, and θ_a_ is the advancing contact angle of the electrolyte, which is usually material‐dependent.^[^ ^6^, ^13^ ^]^ The typical advancing contact angle on a copper surface is around 110°; this value can further increase with increasing surface roughness.^[^ ^16^, ^17^ ^]^ We note that Wu et al. have previously shown that carbon gas diffusion electrodes with microporous layers containing smaller pores exhibit higher contact angles, which indicate better hydrophobicity.^[^ ^18^ ^]^

Herein, we have designed a hydrophobic Cu catalyst layer with a high density of mesopores, and found it very active for CORR (Scheme 1a,b). We first synthesized nanoneedle‐like CuO by using the templating effect of alkali metal K^+^ and Cs^+^ ions. These catalysts were then loaded onto carbon GDEs, and used for CORR. The oxide‐derived (OD) Cu catalyst layers synthesized using K^+^ and Cs^+^ templates exhibited better CORR performance than “unmodified” OD‐Cu. Pore size measurements and contact angle tests of the catalysts unveiled the correlation between pore size and hydrophobicity of the electrodes. We propose that the enhanced hydrophobicity of a catalyst layer allows more triple‐phase (CO‐electrolyte‐catalyst) interfaces to be available for CORR.

## 2 Results and Discussion

### 2.1 Synthesis and Characterization of Cu Catalyst Layers

1D nanomaterials, such as nanoneedles and nanowires are more prone to form 3D porous structures, owing to their inherent anisotropic morphology.^[^ ^19^, ^20^, ^21^ ^]^ This is also likely because during the fabrication of the CL, the nanoneedle deposited first prevents the subsequent nanoneedles from being tightly stacked, thus making it easier to form a porous layer.^[^ ^22^, ^23^ ^]^ Zou et al. and Novellani et al. have further shown, through mathematical models and experiments, that when particles have larger aspect ratios, the resulting packing layer tends to have a higher porosity.^[^ ^24^, ^25^ ^]^ In the field of electrospinning, theoretical models and experimental studies have both shown that the diameter of nanofibers significantly influences the pore size, with smaller nanofiber diameters leading to smaller pores in the electrospun layer.^[^ ^26^ ^]^

On the basis of the above discussion, we first synthesize nanoneedle‐like copper oxides using a chemical coprecipitation method (Scheme 1c; and Supporting Information S1.1). Potassium nitrate (KNO_3_) and caesium nitrate (CsNO_3_) were used to influence the growth of CuO nanomaterials. The Cs^+^ and K^+^ adsorbed with different energies on the crystal faces, which causes the nanoparticles to grow in specific directions and thus have different aspect ratios.^[^ ^27^, ^28^ ^]^ These materials are termed as CuO‐Cs and CuO‐K. CuO grown without the addition of the alkali metal ions was also prepared as the control sample. We note that organic surfactants, such as polyvinylpyrrolidone (PVP) and cetyltrimethylammonium bromide (CTAB) are often used to tune the growth of nanoparticles.^[^ ^29^, ^30^ ^]^ However, these tend to remain, post‐synthesis, as organic residues on the surface of the particles.^[^ ^31^ ^]^ In contrast, alkali metal nitrates are extremely soluble in water and can be removed easily.

**Figure 1:**
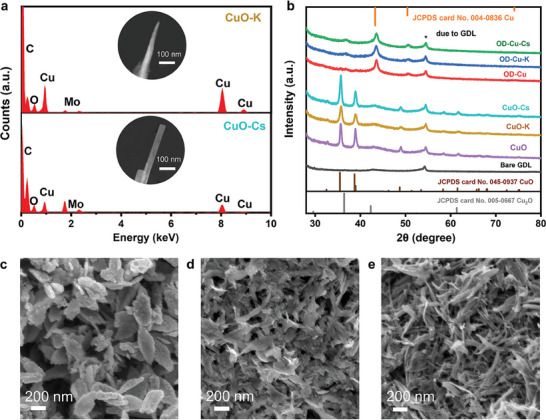
a) EDS of CuO‐K and CuO‐Cs and their corresponding TEM images. b) XRD patterns of freshly‐prepared CuO, CuO‐K, and CuO‐Cs, and after their use as catalysts for CORR. Top‐view SEM images of the c) OD‐Cu, d) OD‐Cu‐K, and e) OD‐Cu‐Cs layers.

High‐resolution transmission electron microscopy (HRTEM) shows that with the addition of K^+^ and Cs^+^, the nanoparticles transited from spindle‐ to rectangular nanoneedle‐shaped (**Figure** **1**a; and Figure S2, Supporting Information). The aspect ratios of the particles also increased significantly, from ∼2.4 in CuO to ∼7.2 in CuO‐K, and further to ∼12.5 in CuO‐Cs. Energy‐dispersive spectroscopy (EDS) of the freshly‐prepared CuO‐K and CuO‐Cs showed the absence of K and Cs signals; the alkali metal cations were likely removed when the catalysts were rinsed during their preparation (Section S1.1, Supporting Information).

To make the proof‐of‐concept that the porosity of a Cu catalyst layer is related to its hydrophobicity, we first spray‐coat the as‐prepared CuO, CuO‐K, and CuO‐Cs onto carbon gas diffusion layers (GDL). X‐ray diffraction (XRD) revealed that these three materials exhibited Cu^II^O diffraction peaks (Figure 1b). After being electrochemically reduced at −20 mA cm^−2^ for 20 min, the oxide‐derived (OD) CLs gave characteristic diffraction peaks of Cu metal. A broad diffraction peak associated with cuprous oxide (Cu_2_O) was also observed at 36.5°, which may be attributed to the reoxidation of metallic Cu once the negative potentials were removed.^[^ ^32^ ^]^

To measure the pore structure in the CLs, we also sprayed catalyst inks onto 3 × 3 cm^2^ flat copper foils (catalyst loading = 10 mg cm^−2^). These as‐prepared electrodes were similarly reduced at −20 mA cm^−2^ for 20 min to obtain OD‐Cu, OD‐Cu‐K, and OD‐Cu‐Cs CLs. Scanning electron microscopy coupled with image analysis of the OD‐Cu‐Cs and OD‐Cu‐K layers show that they have pronounced porous structures, which span two scale levels, namely, meso‐ and macro‐pores (mesopores and macropores are pores with respective diameters of 2–50 and >50 nm; Figure 1c–e).^[^ ^33^ ^]^ The OD‐Cu‐Cs layer has more abundant mesopores. The number of mesopores is as high as 39% in the OD‐Cu‐Cs layer (Figure S3, Supporting Information). In contrast, the OD‐Cu layer has mainly macropores that are larger than 200 nm in size.

**Figure 2:**
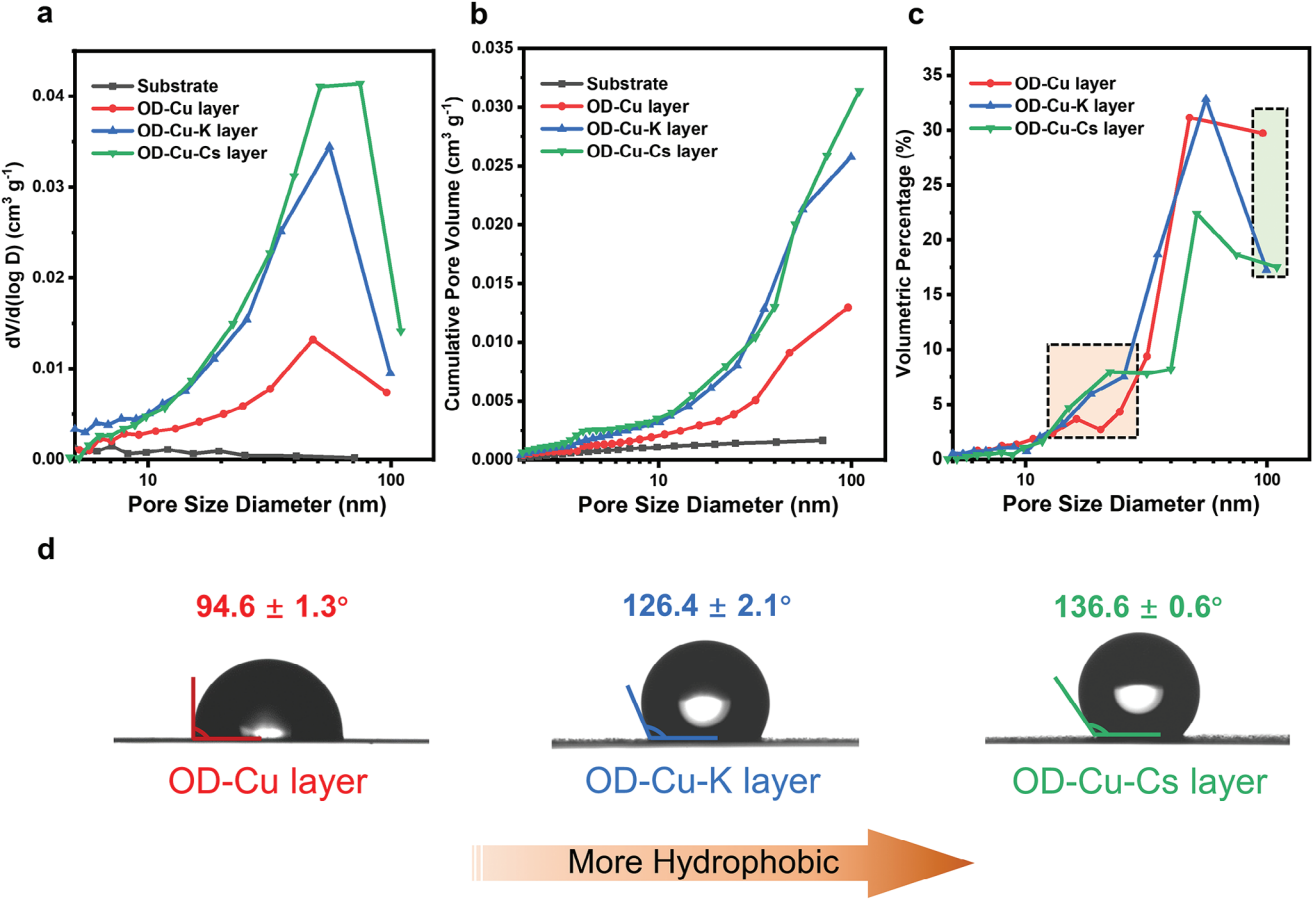
a) Pore size distribution of OD‐Cu, OD‐Cu‐K, and OD‐Cu‐Cs catalyst layers. b) Cumulative pore volume of catalyst layers formed by OD‐Cu, OD‐Cu‐K, and OD‐Cu‐Cs. c) The volumetric percentage of different‐sized pores to the cumulative pore volume for different catalyst layers; the orange and green boxes are representative mesopore and macropore ranges, respectively. d) Contact angles of OD‐Cu, OD‐Cu‐K, and OD‐Cu‐Cs layers after they were subjected to CORR at −20 mA cm^−2^ for 20 min.

Nitrogen physisorption measurement was further employed to analyze the pore size distribution and porosity of the CLs. The nitrogen isotherms are modeled by the Barrett–Joyner–Halenda (BJH) method to give the pore size distribution and porosity of the CLs (Figure S4, Supporting Information).^[^ ^34^ ^]^ The OD‐Cu‐Cs layer exhibited the most abundant mesoporosity in terms of pore volume, followed by the OD‐Cu‐K layer, OD‐Cu layer, and finally, the Cu foil substrate (**Figure** **2**a). The OD‐Cu‐Cs layer exhibits a peak at ∼79 nm, indicating that while it possesses a rich mesoporous structure, it also features small macropores. This indicated that the OD‐Cu‐Cs layer has a hierarchical porous architecture, in good agreement with the results obtained using SEM. The mean free path (MFP) of CO molecules is ∼65 nm at 25 °C and 1 bar.^[^ ^35^ ^]^ Therefore CO transfer in mesopores will rely on Knudsen diffusion, which may lead to a low CO diffusion coefficient and local concentration.^[^ ^10^ ^]^ The presence of smaller macropores in the OD‐Cu‐Cs CL ameliorates these deficiencies. Overall, we highlight that while macropores in a hierarchal porous structure will facilitate CO mass transfer, they also allow electrolyte flooding, as observed in previous works.^[^ ^36^ ^]^ On the other hand, the mesopores will concurrently mitigate the issue of electrolyte infiltration. Therefore, balancing the distribution of pore sizes in a catalyst layer is crucial.

We further found that the cumulative pore volume of the OD‐Cu‐Cs layer is >2× that of the OD‐Cu layer (0.031 vs 0.012 cm^3^ g^−1^), which implies the former's higher porosity at the same mass loading (Figure 2b). In addition, the average BET surface areas for OD‐Cu‐Cs and OD‐Cu‐K are 4.3 and 3.8 m^2^ g^−1^, respectively, which is higher than that of OD‐Cu at 2.2 m^2^ g^−1^. These trends show that a high porosity is accompanied by a high CL surface area. The pore size distribution curve of the Cu substrate itself is almost flat, which indicates that it is nonporous, as expected.

The contribution of different‐sized pores to the cumulative pore volume is shown in Figure 2c. OD‐Cu‐Cs and OD‐Cu‐K layers have a higher volumetric share than OD‐Cu layer at the mesopore range, especially in the orange‐coloured region. Comparing the volumetric share of the largest pores that can be detected, OD‐Cu layer has up to 30%, while OD‐Cu‐Cs and OD‐Cu‐K layers have only 17%, as shown in the green region. This indicates that a large proportion of the cumulative pore volume of OD‐Cu layer is contributed by larger macropores, instead of mesopores.

We next assess the hydrophobicity of the catalyst layers using contact angle tests (Figure 2d). The contact angles of freshly‐prepared CuO, CuO‐K, and CuO‐Cs layers were 125.9°, 133.5°, and 140.6°, respectively (Figure S5, Supporting Information). After being subjected to CO electrolysis at −20 mA cm^−^^2^ for 20 min, their contact angles were 94.6°, 126.4°, and 136.6°, respectively. By comparing the contact angles before and after the CORR, the hydrophobicity of OD‐Cu‐Cs and OD‐Cu‐K only slightly decreased by ∼5°, whereas OD‐Cu significantly lost its hydrophobicity by ∼30°. This may be attributed to the difference in structural stability of the three CLs, i.e., the agglomeration of OD‐Cu particles during CORR probably led to a further increase in macropores.

All in all, we have illustrated the concept that in a copper CL, the smaller its pore sizes, the more hydrophobic it is. We suggest that the small pores (mainly mesopores) within the CL can act as a barrier against electrolyte flooding.

### 2.2 Electrochemical CORR

#### 2.2.1 Linear Sweep Voltammetry in N_2_‐ and CO‐Saturated Electrolytes

We now test the CORR activity of the three Cu GDE in flow cells (Section S3, Supporting Information). The catalysts (2 mg cm^−2^) were first pre‐reduced at −3 A cm^−2^ for 45 min in CO‐saturated 1 m KOH electrolyte. Their catalytic activities were then investigated using linear sweep voltammetry (LSV) in N_2_‐ and CO‐saturated 1 m KOH electrolyte.

**Figure 3:**
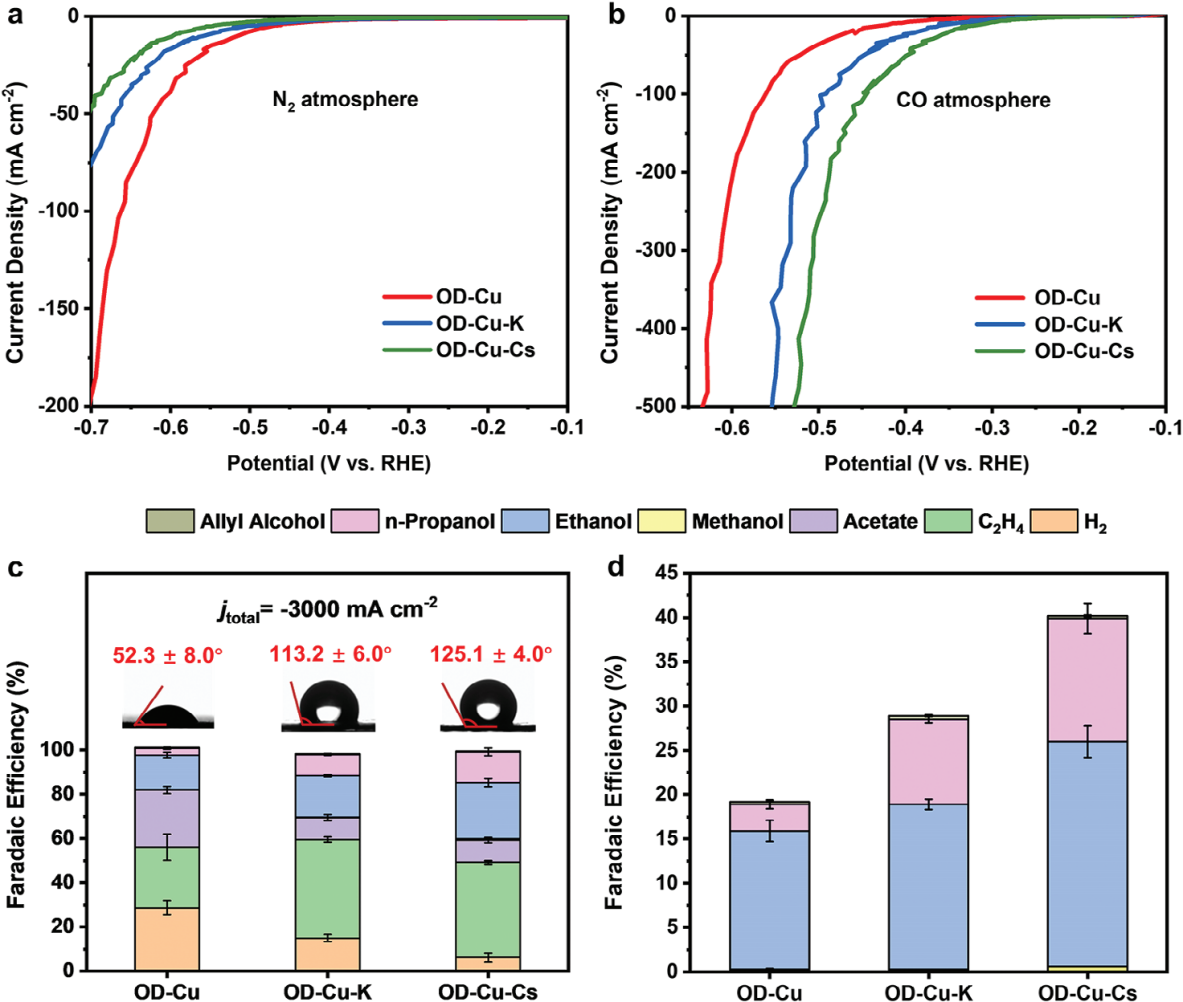
LSVs of OD‐Cu, OD‐Cu‐K, and OD‐Cu‐Cs in a) N_2_‐ and b) CO‐ saturated 1 m KOH electrolyte. Scan rate: 5 mV s^−1^. The mass loadings of CuO, CuO‐K, and CuO‐Cs are 2 mg cm^−2^. The current interrupt mode was used for IR‐drop correction. c) Comparison of the performances of catalysts for CORR at −3 A cm^−2^. Inset: contact angles of the GDEs after they were used for CORR. d) Comparison of the *FE*_alcohols_ exhibited by the different catalysts during CORR at −3 A cm^−2^. All the above‐mentioned measurements were done in a flow cell electrolyzer. 1 m KOH was used as electrolyte.

Under N_2_‐saturation, where the parasitic hydrogen evolution reaction (HER) is expected to occur, OD‐Cu showed the highest HER activity, while OD‐Cu‐Cs showed the lowest (**Figure** **3**a). For example, OD‐Cu and OD‐Cu‐Cs respectively exhibited current densities of −195 and −47 mA cm^−2^ at −0.7 V versus reversible hydrogen electrode (RHE). This observation indicates that OD‐Cu‐Cs has a lower HER activity, or it is more hydrophobic. The three catalysts showed opposite trends in CO‐atmosphere, with OD‐Cu‐Cs having the highest current density (−500 mA cm^−2^ at −0.53 V vs RHE; Figure 3b).

We further normalized the currents obtained from the aforementioned LSV curves to the electrochemically active surface areas (ECSA) of the catalysts (Figures S6, S7 and Table S1, Supporting Information), and found similar trends as shown in Figure 3. This suggests that the electrochemical behaviors of the three catalysts cannot be simply attributed to differences in their ECSAs.

#### 2.2.2 Constant Current CO Electrolysis

We next performed constant current CO electrolysis at −3 A cm^−2^ in 1 m KOH using the OD‐Cu‐Cs, OD‐Cu‐K, and OD‐Cu catalyst layers (2 mg cm^−2^) (Figure 3c; and Table S2, Supporting Information). The duration of the electrolysis was 45 min. The FE of H_2_ in descending order is OD‐Cu with the highest HER (29%), followed by OD‐Cu‐K (15%), and OD‐Cu‐Cs (6%). This finding agrees with the LSV data (Figure 3a). OD‐Cu‐Cs and OD‐Cu‐K exhibit alcohols’ (methanol, ethanol, *n*‐propanol, and allyl alcohol) FEs of 40% and 29%, respectively, significantly surpassing OD‐Cu's 19% (Figure 3d). The ethylene FEs on OD‐Cu‐Cs and OD‐Cu‐K are similar at 43% and 45%, respectively, much higher than that of OD‐Cu at 27%.

Interestingly, we found that OD‐Cu showed a higher acetate FE of 26%, as compared to OD‐Cu‐K and OD‐Cu‐Cs (both FEs = 10%). Acetate is generally considered to be formed by the reaction of water with ethenone intermediates (CH_2_CO).^[^ ^37^ ^]^ We propose that OD‐Cu‐K and OD‐Cu‐Cs GDEs may be less susceptible to electrolyte flooding at high current densities. Consequently, their microenvironment has less water, which thus reduces acetate formation. To further verify the hydrophobicity of the electrodes, we performed contact angle tests on the GDEs after their use for CO electrolysis and found that the OD‐Cu‐Cs layer still has excellent hydrophobicity even after electrolysis at −3 A cm^−2^. The contact angle of OD‐Cu‐Cs is 125.1° and OD‐Cu‐K layer is 113.2°, while OD‐Cu layer has lost its hydrophobicity and its contact angle is only 52.3° (Insets in Figure 3c).

**Figure 4:**
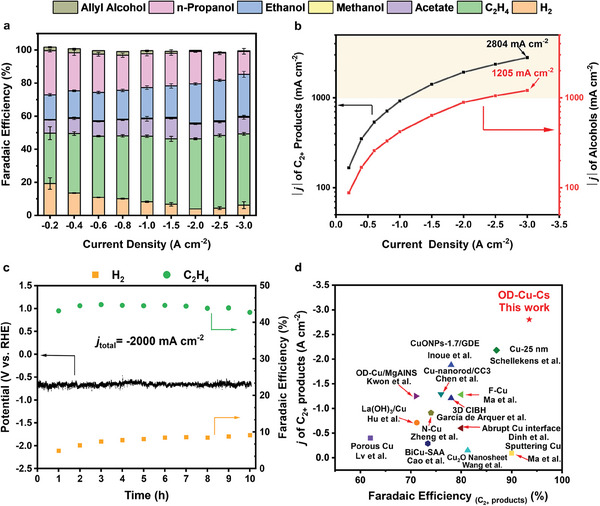
a) Faradaic efficiencies of the CORR products formed at various applied current densities on OD‐Cu‐Cs/GDE in 1 m KOH electrolyte. Reaction time: 45 min. b) Partial current densities of C_2+_ products and alcohols formed at various total current densities using the OD‐Cu‐Cs. c) Stability test of OD‐Cu‐Cs during constant current CO electrolysis at −2000 mA cm^−2^. d) Comparison of the FE_C2+_ and j_C2+_ for OD‐Cu‐Cs (Mass loadings of CuO, CuO‐K, and CuO‐Cs are 2 mg cm^−2^; this work) with that of previously‐reported Cu‐based CO_2_RR and CORR catalysts in alkaline solutions. References: Porous Cu by Lv et al.,^[^ ^41^ ^]^ OD‐Cu/MgAlNS by Kwon et al.,^[^ ^42^ ^]^ La(OH)_3_/Cu by Hu et al.,^[^ ^43^ ^]^ BiCu‐SAA by Cao et al.,^[^ ^44^ ^]^ N‐Cu by Zheng et al.,^[^ ^45^ ^]^ Cu‐nanorod/CC3 by Chen et al.,^[^ ^46^ ^]^ 3D CIBH by García de Arquer et al.,^[^ ^47^ ^]^ CuONPs‐1.7/GDE by Inoue et al.,^[^ ^48^ ^]^ F‐Cu by Ma et al.,^[^ ^49^ ^]^ Abrupt Cu interface by Dinh et al.,^[^ ^50^ ^]^ Cu_2_O nanosheets by Wang et al.,^[^ ^51^ ^]^ Sputtering Cu by Ma et al.,^[^ ^52^ ^]^ and Cu‐25 nm by Schellekens et al.^[^ ^53^ ^]^ All measurements were done in a flow‐cell electrolyzer, with CO_(2)_ flowing into the cell. More details are available in Table S4 (Supporting Information).

We next assess the CORR performance of OD‐Cu‐Cs (the best‐performing CORR electrode) from −200 to −3000 mA cm^−2^ (**Figure** **4**a; and Table S3, Supporting Information). We found that as the applied current density increased, the FE of H_2_ decreased from 19% at −200 mA cm^−2^ to 4% at −2000 mA cm^−2^, and then increased to 6% at −3000 mA cm^−^^2^. At −200 mA cm^−^^2^, OD‐Cu‐Cs exhibits a 27% FE for *n*‐propanol, which is one of the highest values reported in the literature for CO_(2)_RR using copper.^[^ ^38^, ^39^ ^]^ As the current density increases, the FEs of ethanol and ethylene start to increase, and the FE of *n*‐propanol decreases to 14%. Jouny et al. have suggested that this may be due to the second C*─*C coupling step of *n*‐propanol formation being more sluggish compared to the protonation reaction of the C_2_ intermediates at high current densities.^[^ ^40^ ^]^ In terms of total alcohols’ FEs, 40%–45% was maintained at all current densities. Overall, OD‐Cu‐Cs maintains excellent CORR activity over a wide range of current densities; the highest C_2+_ FE was 96% achieved during CO electrolysis at −2 A cm^−2^ (−0.66 V vs RHE). The partial current densities of C_2+_ products and total alcohols produced at different applied current densities are shown in Figure 4b. The highest partial current density of C_2+_ products and total alcohols reached −2804 and −1205 mA cm^−2^, achieving ampere current‐level breakthrough. It is noteworthy that the OD‐Cu‐Cs catalyst layer had exhibited excellent selectivity for CORR products, indicating that sufficient hydrogenation could occur, despite the catalyst being highly hydrophobic.

We assessed the stability of OD‐Cu‐Cs during CORR in a flow cell using 1 m KOH at −2 A cm^−2^ (Figure 4c). The $FE_{H_{2}}$ increased from 5% in the 1st hour to 9% in the 10th hour, while FE_C2H4_ is maintained throughout above 40%. This attests to the superior C_2+_ selectivity and stability of OD‐Cu‐Cs even at high current densities. We note that CO_(2)_RR stability tests over −1 A cm^−2^ have rarely been reported. Inoue et al. reported a *j*_total_ = −1600 mA cm^−2^ stability tests of CO_2_RR in 1 m KCl and found that the selectivity of C_2+_ products decayed to 60% after 6 hours.^[^ ^48^ ^]^ Ma et al. reported a rapid decrease in FE of C_2+_ products from ∼86% to ∼45% within 1 hour when applying −1200 mA cm^−^^2^ without replacing the GDE.^[^ ^49^ ^]^ It is generally believed that GDEs suffer from hydrophobicity losses and thus flood over time. Herein, we show that the mesopore‐rich structure of OD‐Cu‐Cs layer slowed down the electrolyte flooding process, thus allowing it to exhibit excellent CORR selectivity and stability (Figure 4d; and Table S4, Supporting Information).

Finally, we conducted constant potential electrolysis on the three catalysts (Table S5, Supporting Information). The data and trends in the types of products formed are similar to those obtained using constant current electrolysis.

Extensive research has been conducted on how oxide‐derived Cu catalyses CO_(2)_RR.^[^ ^54^, ^55^ ^]^ Our results indicated that the CuO starting material reduced largely to metallic Cu during CORR (Figure 1b), in agreement with previous works.^[^ ^56^ ^]^ Furthermore, the OD‐Cu‐Cs catalyst has morphologies that resemble needles, indicating the presence of highly undercoordinated sites known for catalysing CORR.^[^ ^57^ ^]^ However, we are not able to rule out if minute amounts of O atoms could be present in the catalyst layer during electrolysis, and how these might impact the CORR activity and selectivity.^[^ ^58^ ^]^

The current consensus is that CO is a key intermediate in forming C_2+_ products.^[^ ^59^, ^60^ ^]^ The coverage of CO on a catalyst (𝜃_co_) is thus pivotal in influencing the formation and selectivity of C_2+_ products.^[^ ^61^ ^]^ We note that this might explain why OD‐Cu showed a rather different product selectivity during CO electrolysis at high current densities (−3 A cm⁻^2^), in comparison to OD‐Cu‐K and OD‐Cu‐Cs: the higher extent of electrolyte flooding for OD‐Cu could have limited CO mass transport to its catalyst surface, thus significantly altering its 𝜃_co_. We also note that all three catalysts exhibited similar Tafel slopes for the formation of C_2+_ products (∼130 mV dec⁻¹), which align with the previous report by Lu et al. (Figure S8, Supporting Information).^[^ ^62^ ^]^

In this work, we have demonstrated a less‐is‐more electrode design concept; we illustrate the feasibility of incorporating hydrophobicity into CLs by solely relying on their structural architectures, rather than by employing exogenous additives, such as PTFE. This work provides a clear picture of the impact of the microstructure of the CL, such as the pore size distribution, on the CORR performance. By changing the morphology of the catalyst to influence the structure of the formed CLs, the active sites of the catalyst are exposed as much as possible while obtaining good hydrophobicity. The enhanced hydrophobicity will also allow more triple‐phase interfaces to be available for CORR. Consequently, HER is minimized, while CO electro‐conversion to −2.8 A cm^−2^ C_2+_ products is enabled (Figure 4d). Our work further reveals that outstanding CORR performances can actually be obtained by only using copper in the catalyst layer, in comparison to other recent works where dopants, such as silver and cationic surfactants had to be added to obtain a performance boost.^[^ ^63^, ^64^, ^65^ ^]^

## 3 Conclusion

Herein, we present a strategy to increase the hydrophobicity of electrodes by relying on the mesopore‐rich structure of a catalyst layer. We do so by modifying the growth of CuO nanoparticles through the template effect of alkali metal cations to increase their aspect ratios. Consistent with results from SEM imaging and nitrogen physisorption measurements, the CLs formed by these nanomaterial building blocks are especially rich in mesopores, as compared to “unmodified” OD‐Cu CL. Contact angle tests indicate that the hydrophobicity is in descending order of OD‐Cu‐Cs, OD‐Cu‐K, and OD‐Cu layer, which suggests a positive correlation between hydrophobicity and mesopore richness of CLs. OD‐Cu‐Cs has the best CORR activity, and highest C_2+_ FE up to 96% at −2 A cm^−2^. ∼90% C_2+_ FE at −2 A cm^−2^ was maintained even after 10 hours. Even during CO electrolysis at −3 A cm^−2^, the OD‐Cu‐Cs still demonstrates outstanding partial current densities of −2804 and −1205 mA cm^−2^ for C_2+_ products and total alcohols, respectively. These values represent one of the best CORR figures‐of‐merit in all reported literature. Our work highlights the importance of the microenvironment of the CL, reveals the relationship between pore size and hydrophobicity, and provides directions for electrode design to achieve high current density.

## 4 Experimental Section

Detailed experimental procedures can be found in Sections S1–S3 of the Supporting Information.

## Conflict of Interest

The authors declare no conflict of interest.

## Supporting information

Supporting Information

## Data Availability

The data that support the findings of this study are available from the corresponding author upon reasonable request.
