# Supplementary material for: Electroreduction of CO to 2.8 A cm⁻2 C2+ Products: Maximizing Efficiency with Minimalist Electrode Design Featuring a Mesopore‐Rich Hydrophobic Copper Catalyst Layer
Source: Adv Sci (Weinh). 2024 Aug 26;11(40):2405938. doi: 10.1002/advs.202405938 (PMC11516069; doi:10.1002/advs.202405938)
Supplement: Supplementary file 1 — Supporting Information [file ADVS-11-2405938-s001.docx]

Supporting Information

**Electroreduction of CO to 2.8 A cm⁻² C_2+_ Products: Maximizing Efficiency with Minimalist Electrode Design Featuring a Mesopore-Rich Hydrophobic Copper Catalyst Layer**

Silu Chen^1^, Ben Rowley^2^, Ramesha Ganganahalli^3^, and Boon Siang Yeo^1,*^

^1^ Department of Chemistry, National University of Singapore, 3 Science Drive 3, Singapore 117543

^2^ Energy Transition Campus Amsterdam, Grasweg 31, 1031 HW, Amsterdam, The Netherlands

^3^ Shell India Markets Private Ltd. Plot No. 7, Bengaluru Hardware Park, Mahadeva, Kodigehalli, Bangalore, North 562149, India

**S1. Materials and Methods**

**S1.1. Sample preparation**

**Synthesis of CuO, CuO-K and CuO-Cs:** CuO was synthesized using a chemical co-precipitation method:^[1]^ 30 ml of an aqueous 1 M copper nitrate trihydrate (Cu(NO_3_)_2_∙3H_2_O, ≥98 %, Sigma-Aldrich) solution was made, and stirred for 30 min. Then, with vigorous stirring, 5 M aqueous sodium hydroxide (NaOH, ≥99.8%, GCE) was slowly added until the pH reached 10. The solution was then heated at 90 °C for 5 hours in an oil bath. At the end of 5 hours, the flask was raised from the oil bath and naturally cooled to room temperature (~25℃). The obtained nanomaterials were collected by centrifugation and washed with DI water and ethanol four times each to remove any unreacted precursors. The washed precipitates were dried at 90 °C for 12 hours in a hot air oven (BINDER GmbH, Germany).

For the preparation of CuO-K and CuO-Cs, we have employed the same procedure as for CuO, except that 1.58 mmol potassium nitrate (KNO_3_, ≥ 99.999 %, Sigma-Aldrich) and caesium nitrate (CsNO_3_, ≥ 99.999 %, Sigma-Aldrich) were respectively added to the initial copper nitrate trihydrate solution. The prepared CuO nanomaterials were denoted as CuO-K and CuO-Cs. To ensure reproducibility, three separate batches of each catalyst were prepared independently and studied.

**Preparation of electrodes:** To construct the working electrode, a catalyst ink that contained 10 mg of the as-synthesized CuO, CuO-K or CuO-K nanoparticles, 1 mL of isopropanol and 25 µL of Nafion 117 (5 wt. % in lower aliphatic alcohols and water, Sigma-Aldrich) were first mixed and sonicated to give a homogenous solution. Next, the catalyst ink was slowly spray-coated on carbon gas diffusion layers (YLS-30T, Toray Industries Inc., 1.5 cm × 1.5 cm) to achieve a catalyst loading of 2.0 mg cm^−2^. The freshly coated gas diffusion electrode (GDE) was placed under a heat lamp (~75℃) until completely dry. A 3 cm × 3 cm porous nickel foam (Sinero) was used as anode.

**S2. Materials Characterization**

**Characterization of catalysts:** The compositions of the catalyst were determined using X-ray diffraction (XRD; Bruker D8 Advance; Cu Kα 40 kV, 40 mA). The surface morphologies of the catalysts were characterized using scanning electron microscopy (SEM JEOL JSM 6710F), operated in secondary electron mode (5.0 kV). Transmission electron microscopy (TEM) was conducted using a JEM-2200FS at 200kV. Energy-dispersive X-ray spectroscopy (EDX) was performed by using an attached [EDX spectrometer](https://www-sciencedirect-com.libproxy1.nus.edu.sg/topics/engineering/energy-dispersive-x-ray-spectrometer) (Oxford Instruments, X-Max 80T). The catalysts were loaded onto molybdenum grids coated with carbon supporting films (Precise, 300 mesh).

**Contact angle measurement:** The contact angles were measured using a contact angle system (OCA 50 Micro, Dataphysics, Germany) at ~25℃, with the probe liquid being 2 μL of 1 M KOH. All contact angle images were taken when they reached static after the application of the liquid droplet on the surface of the samples. The reported contact angle was obtained by averaging the values taken on three different samples.

**Measurements of pore size distribution and porosity in catalyst layers:** The nitrogen physisorption tests were conducted on a TriStar™ II 3020 (Micromeritics, USA). To ensure sufficient quality for measurement, the CuO, CuO-K and CuO-Cs catalyst inks were respectively sprayed on 3 cm × 3 cm copper foils to give catalyst loadings of 10 mg cm^-2^. The as-prepared electrodes were then reduced at a current density of -20 mA cm^-2^ for 20 min to obtain OD-Cu, OD-Cu-K and OD-Cu-Cs layers. The reduced electrodes (including the substrate) were cut into approximate 1.5 cm × 0.5 cm strips to fit into the sample tubes. These samples were degassed at 120 °C for 12 hours prior to the physisorption experiment. The nitrogen physisorption experiments were performed immediately after degassing. Subsequently, the nitrogen isotherms are modelled by the Barrett-Joyner-Halenda (BJH) method to achieve pore size distribution and pore volume of samples. The test was repeated three times to verify its repeatability.

**S3. Electrochemical measurement**

**Electrocatalytic reduction of CO:** CORR was performed using a 3-electrode configuration in a flow cell electrolyzer (Shanghai Chuxi Industrial Co.; Figure S1). The anodic and cathodic chambers were separated by an anion−exchange membrane SELEMION™ (AMV, Asahi Glass). A Ag/AgCl (saturated KCl; Pine Research Instrumentation) was used as a reference electrode, and a 3 cm × 3 cm porous Ni foam (Sinero) was used as a counter electrode. The exposed geometric surface area of the working electrode was 1 cm × 1 cm. The flow cell was circulated with aqueous 1 M KOH (Meryer, 99.999%) electrolyte using a peristaltic pump. CO gas (99.97%, Linde) was flowed into the cell at a flow rate of 40 sccm. A Gamry Reference 3000™ potentiostat was used to control the electrochemistry. The gaseous and volatile liquid (in both the anolyte and catholyte) products were analyzed and quantified using an online gas chromatograph (GC, Agilent 8890; FID and TCD) and a headspace gas chromatograph (Headspace GC, Agilent 7890B and 7697A; FID). Acetate was analysed and quantified by a high-performance liquid chromatograph (HPLC, Agilent 1260 Infinity), with a variable wavelength detector (VWD, detection limit < 5 μM). All reaction times were 45 mins, if not otherwise stated. All current densities reported in this work were normalized to the exposed geometric surface area of the electrode, if not otherwise stated. In this work, all measured potentials were converted to the reversible hydrogen electrode (RHE) scale according to:

E (vs. RHE) = E (vs. Ag/AgCl) + 0.197 V + (0.0591 × pH). (1)

Unless specifically mentioned, the measured potentials were also corrected using:

E (vs. RHE) = E (vs. Ag/AgCl) + 0.197 V + (0.0591 × pH) – 0.85 iR (2)

Where i is the total current. The ohmic loss R between the working and reference electrodes was measured using the electrochemical impedance spectroscopy technique. A factor of 0.85 is used for the correction term.^[2, 3]^

An example of converting E vs. Ag/AgCl to E vs. RHE (E measured during constant current electrolysis at -2 A cm^-^²): the overall average ohmic loss was approximately 1 ohm. Additionally, the potential measured during constant current electrolysis at -2 A cm^-^² was -3.4 V vs. Ag/AgCl.

Substituting into the aforementioned Equation (2):

E (vs. RHE) = −3.4 + 0.197 + (0.0591 × 14) - (0.85 × -2 × 1) = −0.676 V

**Electrochemical surface areas (ECSA) evaluation:** The electrochemical double-layer capacitance method was employed for evaluating the ECSAs of the catalysts.^[4, 5]^ Each catalyst (mass loading = 2 mg cm^-2^) was reduced at −3 A cm^-2^ for 45 min in a flow cell and then scanned in the potential range of 0.2 to 0.3 V vs. RHE in N_2_-saturated 1 M KOH at scan rates of 10, 20, 30, 40, 50 and 60 mV s^−1^. N_2_ gas was flowed into the cathodic compartment during the measurement. The double-layer capacitive current density was plotted against the scan rate of the CV for each catalyst. The slope of the linear regression gives the double-layer capacitance. The double layer capacitance of bare GDE was measured to be 24.7 μF cm^-^², consistent with previous literature values at the microFarad level.^[6]^ Thus, its influence can be neglected. Previous studies have determined the double-layer capacitance of an electropolished copper foil to be 0.029 mF cm^-2^.^[7]^

**4. Supplementary Tables**

**Table S1.** Double layer capacitance and corresponding ECSA for different catalysts.

| **Sample** | **Capacitance C_dl_ (mF cm^-2^)** | | **ECSA^a^ (cm^2^)** |
| --- | --- | --- | --- |
| OD-Cu | 6.9 | 237.9 | |
| OD-Cu-K | 14.6 | 503.4 | |
| OD-Cu-Cs | 22.1 | 762.1 | |

^a^The ECSAs were obtained by dividing the C_dl_ values by 0.029 mF cm^−2^ (C_dl_ of an ideally flat Cu foil).

**Table S2.** The Faradaic efficiencies of COR products formed using OD-Cu, OD-Cu-K and OD-Cu-Cs in a flow cell. Reaction conditions: Constant current electrolysis at – 3000 mA cm^-2^ were performed. 1 M KOH was used as electrolyte. A Ni foam was used as the anode.

| Catalysts | Potential | | FE (%) | | | | | | | | | | | | Total FE (%) |
| --- | --- | --- | --- | --- | --- | --- | --- | --- | --- | --- | --- | --- | --- | --- | --- |
|  | V^[a]^ | V^[b]^ | Hydrogen | Methane | Ethylene | Acetate | Acetaldehyde | Propionaldehyde | Acetone | Methanol | Ethanol | *n*-Propanol | Allyl alcohol | *n*-Butanol |  |
| OD-Cu | - 4.6 ± 0.3 | -0.82 ± 0.04 | 28.7 ± 3.2 | 0.3 ± 0.3 | 27.4 ± 5.9 | 25.8 ± 1.7 | 0.15 ± 0.02 | 0.02 ± 0.01 | 0.15 ± 0.01 | 0.3 ± 0.1 | 15.6 ± 1.2 | 3.0 ± 0.5 | 0.31 ± 0.03 | 0.01 ± 0.01 | 101.8 ± 7.0 |
| OD-Cu-K | - 4.3 ± 0.1 | -0.77 ± 0.02 | 15.0 ± 1.7 | tr | 44.5 ± 1.2 | 10.0 ± 1.4 | 0.19 ± 0.05 | 0.04 ± 0.02 | 0.16 ± 0.01 | 0.32 ± 0.04 | 18.6 ± 0.6 | 9.6 ± 0.4 | 0.4 ± 0.1 | 0.01 ± 0.01 | 98.8 ± 2.7 |
| OD-Cu-Cs | - 3.8 ± 0.4 | -0.73 ± 0.04 | 6.2 ± 2.1 | tr | 43.1 ± 0.8 | 10.0 ± 1.2 | 0.4 ± 0.1 | 0.06 ± 0.02 | 0.27 ± 0.03 | 0.59 ± 0.02 | 25.4 ± 1.8 | 13.9 ± 1.7 | 0.3 ± 0.1 | 0.04 ± 0.01 | 100.2 ± 3. 6 |

tr: trace

[a]: vs. Ag/AgCl

[b]: vs. RHE

**Table S3.** The Faradaic efficiencies of COR products formed using OD-Cu-Cs in a flow cell. Reaction conditions: Constant current electrolysis at various current densities were performed. 1 M KOH was used as electrolyte. A Ni foam was used as the anode.

| *j* | Potential | | FE (%) | | | | | | | | | | | | Total FE (%) |
| --- | --- | --- | --- | --- | --- | --- | --- | --- | --- | --- | --- | --- | --- | --- | --- |
| (mA cm^-2^) | V^[a]^ | V ^[b]^ | Hydrogen | Methane | Ethylene | Acetate | Acetaldehyde | Propionaldehyde | Acetone | Methanol | Ethanol | *n*-Propanol | Allyl alcohol | *n*-Butanol |  |
| -200 | -1.9 ± 0.1 | -0.49 ± 0.02 | 19.3 ± 3.5 | tr | 30.4 ± 3.9 | 8.0 ± 0.2 | 0.59 ± 0.03 | 0.3 ± 0.2 | 0.20 ± 0.04 | 0.32 ± 0.02 | 14.9 ± 0.8 | 26.7 ± 0.8 | 2.1 ± 0.4 | 0.02 ± 0.01 | 102.8 ± 5.3 |
| -400 | -2.3 ± 0.1 | -0.53 ± 0.02 | 13.5 ± 0.2 | tr | 36.0 ± 1.1 | 9.2 ± 0.8 | 0.3 ± 0.1 | 0.1 ± 0.1 | 0.16 ±0.03 | 0.40 ±0.04 | 16.2 ± 0.5 | 23.0 ± 1.6 | 2.4 ± 0.3 | 0.02 ± 0.01 | 101.3 ± 2.3 |
| -600 | -2.51 ± 0.03 | -0.55 ± 0.01 | 10.9 ± 0.2 | tr | 37.0 ± 0.5 | 8.9 ± 0.3 | 0.3 ± 0.1 | 0.1 ± 0.1 | 0.17 ± 0.01 | 0.43 ± 0.05 | 17.1 ± 0.7 | 23.1 ± 1.2 | 2.2 ± 0.1 | 0.02 ± 0.01 | 100.4 ± 1.5 |
| -800 | -2.9 ± 0.2 | -0.57 ± 0.02 | 10.1 ± 0.3 | tr | 38.0 ± 0.8 | 9.7 ± 0.5 | 0.35 ± 0.03 | 0.1 ± 0.1 | 0.18 ± 0.03 | 0.47 ± 0.05 | 17.3 ± 0.8 | 21.3 ± 1.3 | 2.2 ± 0.1 | 0.02 ± 0.01 | 99.8 ± 1.9 |
| -1000 | -3.1 ± 0.3 | -0.59 ± 0.03 | 8.3 ± 0.4 | tr | 39.6 ± 0.8 | 10.5 ± 1.4 | 0.30 ± 0.05 | 0.06 ± 0.02 | 0.19 ± 0.01 | 0.45 ± 0.04 | 18.4 ± 1.1 | 20.5 ± 1.3 | 1.9 ± 0.3 | 0.03 ± 0.01 | 100.1 ± 2.5 |
| - 1500 | -3.21 ± 0.04 | -0.62 ± 0.01 | 6.7 ± 1.1 | tr | 39.6 ± 1.5 | 12.4 ± 0.4 | 0.2 ± 0.1 | 0.1 ± 0.2 | 0.22 ± 0.02 | 0.6 ± 0.1 | 19.0 ± 1.3 | 19.7 ± 1.2 | 1.2 ± 0.9 | 0.1 ± 0.1 | 99.8 ± 2.7 |
| -2000 | -3.3 ± 0.1 | -0.66 ± 0.01 | 3.89 ± 0.04 | tr | 42.4 ± 0.6 | 9.0 ± 0.3 | 0.46 ± 0.01 | 0.08 ± 0.01 | 0.24 ± 0.01 | 0.64 ± 0.02 | 23.6 ± 0.7 | 19.6 ± 0.5 | 0.6 ± 0.1 | 0.05 ±0.01 | 100.6 ± 1.1 |
| -2500 | -3.4 ± 0.1 | -0.69 ± 0.01 | 4.4 ± 0.9 | tr | 43.9 ± 0.7 | 8.3 ± 0.7 | 0.4 ± 0.1 | 0.08 ± 0.03 | 0.29 ± 0.03 | 0.6 ± 0.1 | 24.5 ± 0.7 | 16.4 ± 0.9 | 0.5 ± 0.2 | 0.07 ± 0.04 | 99.4 ± 1.8 |
| -3000 | -3.8 ± 0.4 | -0.73 ± 0.04 | 6.2 ± 2.1 | tr | 43.1 ± 0.8 | 10.0 ± 1.2 | 0.4 ± 0.1 | 0.06 ± 0.02 | 0.27 ± 0.03 | 0.59 ±0.02 | 25.4 ± 1.8 | 13.9 ± 1.7 | 0.3 ± 0.1 | 0.04 ± 0.01 | 100.2 ± 3. 6 |

tr: trace

[a]: vs. Ag/AgCl

[b]: vs. RHE

**Table S4.** The comparison of CO_(2)_RR activity in alkaline electrolytes using Cu-based catalysts in flow cells. The table shows the highest value of *j*_C2+_ and FE_C2+_ under those conditions in each reference.

| **Electrocatalysts** | **Electrolytes** | **FE_C2+_ (%)** | ***j*_C2+_ (A cm^-2^)** | **Reference** |
| --- | --- | --- | --- | --- |
| OD-Cu-Cs | 1 M KOH | 93.5 | -2.8 | This work. |
| Porous Cu | 1 M KOH | 62 | -0.4 | *Adv. Mater.*, **2018**, 30, 1803111.^[8]^ |
| OD-Cu/MgAlNS | 1 M KOH | 71 | -1.25 | *Angew. Chem. Int. Ed.*, **2023**, 62 e202217252.^[9]^ |
| La(OH)_3_/Cu | 1 M KOH | 71.2 | -0.71 | *Small*, **2023**, 20, 23082262.^[10]^ |
| BiCu-SAA | 1 M KOH | 73.4 | -0.29 | *Angew. Chem. Int. Ed.*, **2023**, 62,  e202303048. ^[11]^ |
| N-Cu | 1 M KOH | 74 | -0.91 | *J. Am. Chem. Soc.*, **2022**, 144, 14936-14944.^[12]^ |
| Cu-nanorod /CC3 | 1 M KOH | 76 | -1.29 | *Angew. Chem. Int. Ed.*, **2022**, 61, e202202607.^[13]^ |
| 3D CIBH | 7 M KOH | 78 | -1.21 | *Science*, **2020**, 367, 661-666.^[14]^ |
| CuONPs-1.7/GDE | 1 M KOH | 78 | -1.88 | *EES Catal.*, **2023,** 1, 9-16.^[15]^ |
| F-Cu | 0.75 M KOH | 80 | -1.28 | *Nat. Catal.*, **2020**, 3, 478-487.^[16]^ |
| Abrupt Cu interface | 3.5 M KOH + 5 M KI | 80 | -0.6 | *Science*, **2018**, 360, 783-787.^[17]^ |
| Cu_2_O Nanosheet | 1.0 M KOH | 81.3 | -0.15 | *J. Am. Chem. Soc.*, **2023**, 145, 26133-26143.^[18]^ |
| Sputtering Cu | 1.0 M KOH | 90 | -0.09 | *Energy Environ. Sci.*, **2022**, 15, 2470-2478. ^[19]^ |
| Cu-25 nm | 1.0 M KOH | 87 | -2.18 | *Chem. Eng. J.* **2024**, 483, 149105*.*^[20]^ |

**Table S5.** The Faradaic efficiencies of COR products formed using OD-Cu, OD-Cu-K and OD-Cu-Cs in a flow cell. Reaction conditions: Constant potential electrolysis at various applied potentials were performed. 1 M KOH was used as electrolyte. A Ni foam was used as the anode.

| Potential/  V vs. RHE | Catalyst | *j*_total_/  mA cm^-2^ | FE (%) | | | | | | | | | | | | Total FE (%) |
| --- | --- | --- | --- | --- | --- | --- | --- | --- | --- | --- | --- | --- | --- | --- | --- |
|  |  |  | Hydrogen | Methane | Ethylene | Acetate | Acetaldehyde | Propionaldehyde | Acetone | Methanol | Ethanol | *n*-Propanol | Allyl alcohol | *n*-Butanol |  |
| -0.45 | OD-Cu | -26.5  ± 4.3 | 24.3 ± 1.1 | tr | 26.5 ± 0.5 | 12.8 ± 1.2 | 0.84 ± 0.06 | 1.0 ± 0.1 | 0.20 ± 0.06 | tr | 15.9 ± 2.0 | 21.9 ± 0.3 | 0.99 ± 0.03 | tr | 104.6  ± 2.7 |
|  | OD-Cu-K | -53.7  ± 10.4 | 20.5 ± 4.2 | tr | 25.6 ± 2.0 | 10.2 ± 2.2 | 0.7 ± 0.2 | 0.8 ± 0.2 | 0.10 ± 0.03 | tr | 16.3 ± 0.9 | 26.6 ± 0.2 | 0.6 ± 0.1 | tr | 101.4  ± 5.2 |
|  | OD-Cu-Cs | -69.0  ± 13.9 | 19.3 ± 3.0 | tr | 28.6 ± 1.2 | 7.7 ± 1.1 | 0.5 ± 0.4 | 1.0 ± 0.6 | 0.44 ± 0.53 | tr | 15.2 ± 2.6 | 30.0 ± 2.4 | 1.11 ± 0.36 | tr | 103.9  ± 5.0 |
| -0.50 | OD-Cu | -43.3  ± 2.3 | 23.7 ± 0.6 | tr | 29.8 ± 0.9 | 9.1 ± 1.0 | 1.36 ± 0.06 | 1.2 ± 0.2 | 0.37 ± 0.21 | 0.05 ± 0.09 | 13.6 ± 0.2 | 21.5 ± 0.7 | 1.5 ± 0.1 | tr | 102.2  ± 1.7 |
|  | OD-Cu-K | -116.5 ± 24.3 | 20.1 ± 4.8 | tr | 31.9 ± 3.7 | 8.3 ± 0.8 | 0.8 ± 0.1 | 0.20 ± 0.03 | 0.17 ± 0.03 | 0.2 ± 0.1 | 15.3 ± 1.9 | 22.4 ± 0.8 | 1.3 ± 0.5 | tr | 100.7  ± 6.4 |
|  | OD-Cu-Cs | -233.7 ± 23.5 | 15.7 ± 0.3 | tr | 34.6 ± 0.8 | 8.6 ± 0.8 | 0.63 ± 0.03 | 0.38 ± 0.15 | 0.15 ± 0.02 | 0.3 ± 0.1 | 15.3 ± 0.4 | 24.4 ± 0.9 | 2.0 ± 0.2 | tr | 102.0  ± 1.5 |
| -0.55 | OD-Cu | -110.0 ± 7.3 | 21.3 ± 0.6 | tr | 32.9 ± 2.1 | 11.8 ± 3.6 | 0.85 ± 0.08 | 0.35 ± 0.03 | 0.28 ± 0.06 | 0.24 ± 0.05 | 14.5 ± 0.7 | 18.4 ± 1.3 | 2.3 ± 0.2 | tr | 102.9 ± 4.5 |
|  | OD-Cu-K | -304.9 ± 20.7 | 15.1 ± 0.9 | tr | 37.6 ± 1.6 | 8.6 ± 0.8 | 0.33 ± 0.04 | 0.05 ± 0.01 | 0.10 ± 0.01 | 0.23 ± 0.06 | 14.7 ± 0.5 | 20.5 ± 1.6 | 1.8 ± 0.1 | tr | 99.0 ± 2.6 |
|  | OD-Cu-Cs | -681.3 ± 88.0 | 9.5 ± 1.3 | tr | 40.4 ± 0.8 | 9.3 ± 1.1 | 0.34 ± 0.08 | 0.05 ± 0.01 | 0.31 ± 0.14 | 0.45 ± 0.05 | 18.2 ± 2.0 | 21.7 ± 2.3 | 2.4 ± 0.5 | tr | 102.0 ± 3.6 |
| -0.60 | OD-Cu | -331.0 ± 53.4 | 14.8 ± 0.9 | tr | 35.9 ± 0.8 | 11.6 ± 0.2 | 0.75 ± 0.16 | 0.17 ± 0.08 | 0.23 ± 0.01 | 0.43 ± 0.06 | 18.7 ± 0.2 | 14.6 ± 0.8 | 2.7 ± 0.1 | tr | 99.8 ± 1.5 |
|  | OD-Cu-K | -658.3 ± 79.3 | 9.2 ± 0.3 | tr | 42.4 ± 0.6 | 10.1 ± 1.5 | 0.71 ± 0.09 | 0.83 ± 0.28 | 0.16 ± 0.01 | 0.03 ± 0.01 | 20.19 ± 1.06 | 19.16 ± 0.43 | 2.5 ± 0.3 | tr | 105.4 ± 2.0 |
|  | OD-Cu-Cs | -1386.5 ± 237.5 | 5.9 ± 1.4 | tr | 43.9 ± 1.5 | 8.9 ± 0.4 | 1.0 ± 0.3 | 0.6 ± 0.3 | 0.20 ± 0.05 | 0.3 ± 0.2 | 20.9 ± 2.4 | 20.7 ± 0.3 | 1.6 ± 0.5 | tr | 104.0 ± 3.3 |
| -0.66 | OD-Cu | -694.1  ± 56.1 | 9.6 ± 0.6 | tr | 37.6 ± 0.9 | 12.6 ± 1.1 | 0.29 ± 0.02 | 0.05 ± 0.02 | 0.18 ± 0.02 | 0.38 ± 0.02 | 20.1 ± 1.1 | 16.9 ± 0.5 | 2.05 ± 0.05 | tr | 99.8 ± 2.0 |
|  | OD-Cu-K | -1445.7 ± 111.3 | 9.13 ± 0.38 | tr | 47.1 ± 2.8 | 9.1 ± 2.0 | 0.55 ± 0.04 | 0.22 ± 0.05 | 0.16 ± 0.02 | 0.23 ± 0.06 | 22.18 ± 1.87 | 13.4 ± 1.3 | 0.95 ± 0.07 | tr | 103.1 ± 4.2 |
|  | OD-Cu-Cs | -2243.0 ± 118.5 | 6.48 ± 0.77 | tr | 46.1 ± 0.5 | 9.5 ± 1.5 | 1.10 ± 0.35 | 0.53 ± 0.15 | 0.21 ± 0.03 | 0.37 ± 0.06 | 24.46 ± 2.40 | 14.2 ± 1.1 | 0.53 ± 0.15 | tr | 103.5 ± 3.2 |

tr: trace

**S5. Supplementary Figures**

**
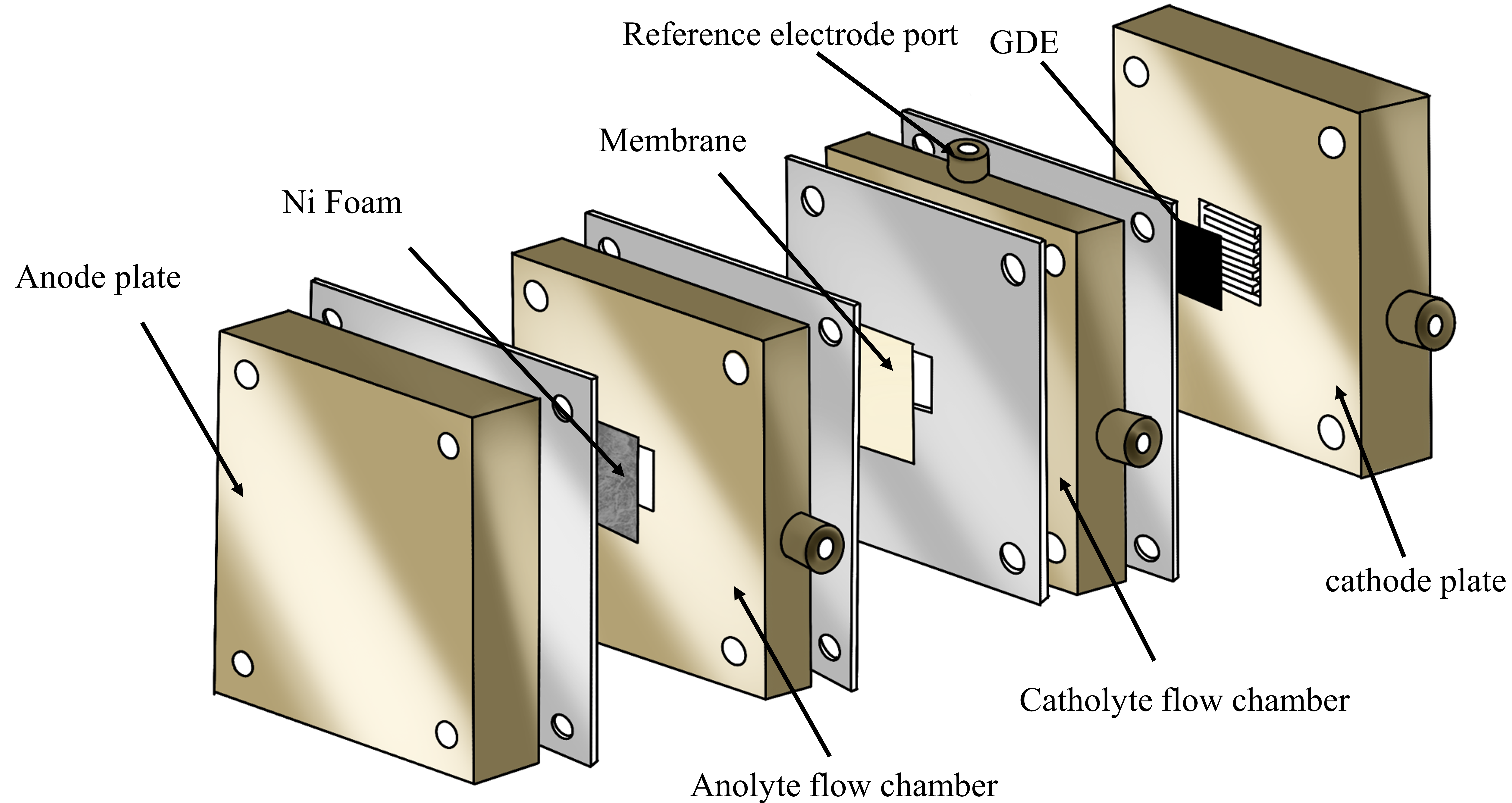
**

**Figure S1**. Schematic diagram of the three-electrodes flow cell used for the CORR.


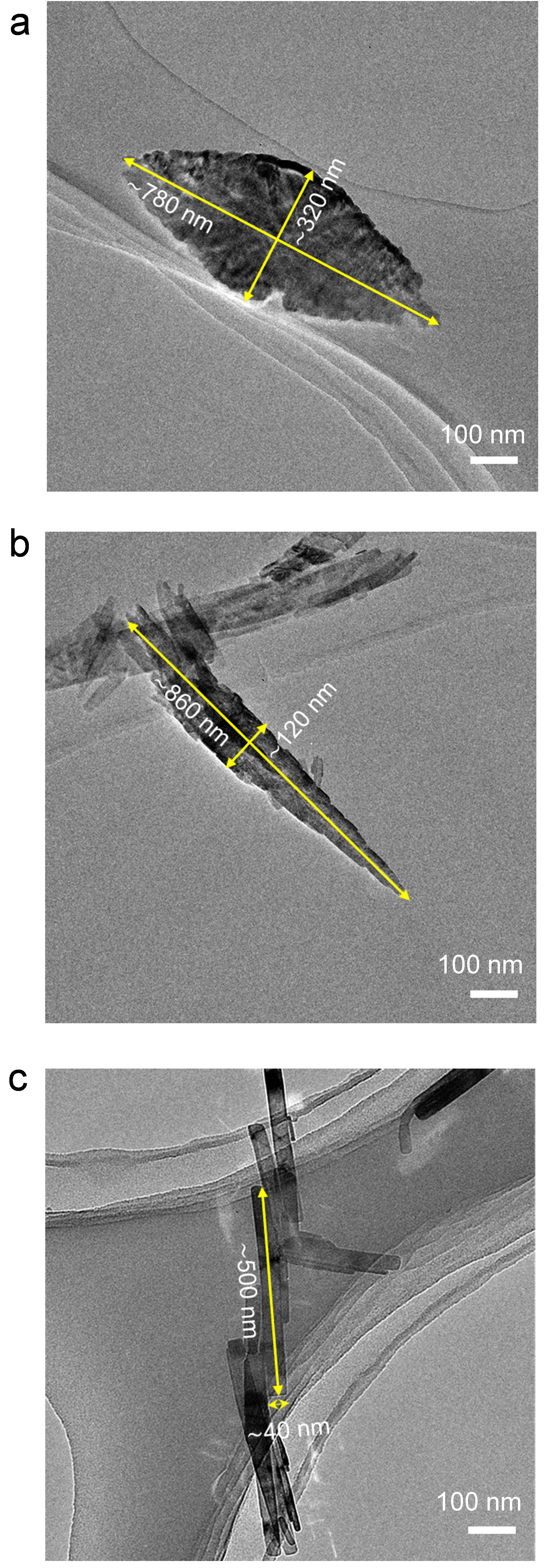


**Figure S2**. High-resolution transmission electron microscopy (HRTEM) images of (a) CuO, (b) CuO-K, and (c) CuO-Cs particles.


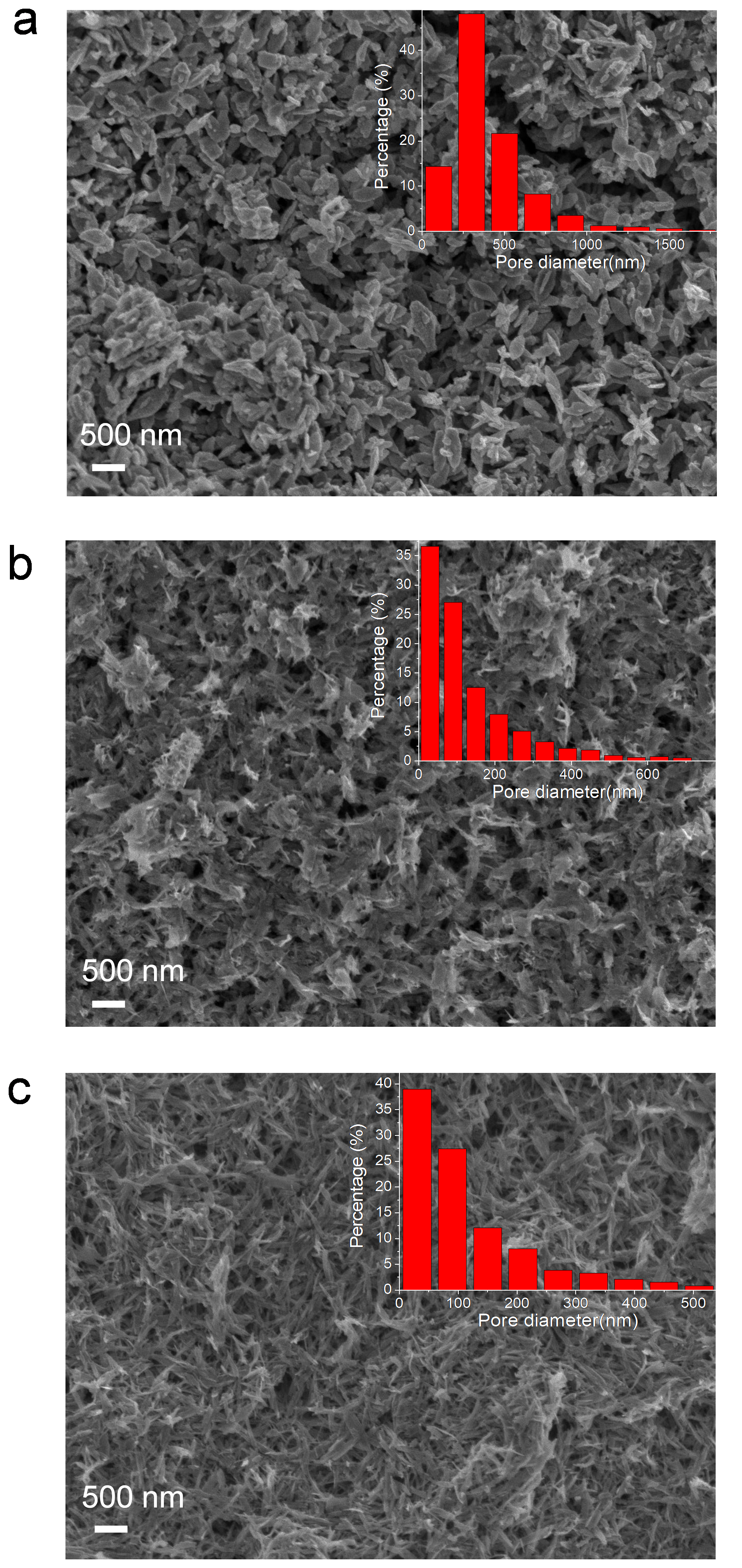


**Figure S3**. Top-view SEM images of (a) OD-Cu, (b) OD-Cu-K and (c) OD-Cu-Cs layers. The top right inset in each figure shows the pore size distribution (%) in the CLs. Percentage (%) =$\frac{The number of pores of specific size}{Total number of pores in the counting area}$. The pores are identified and quantified by the Image J software.

a

d


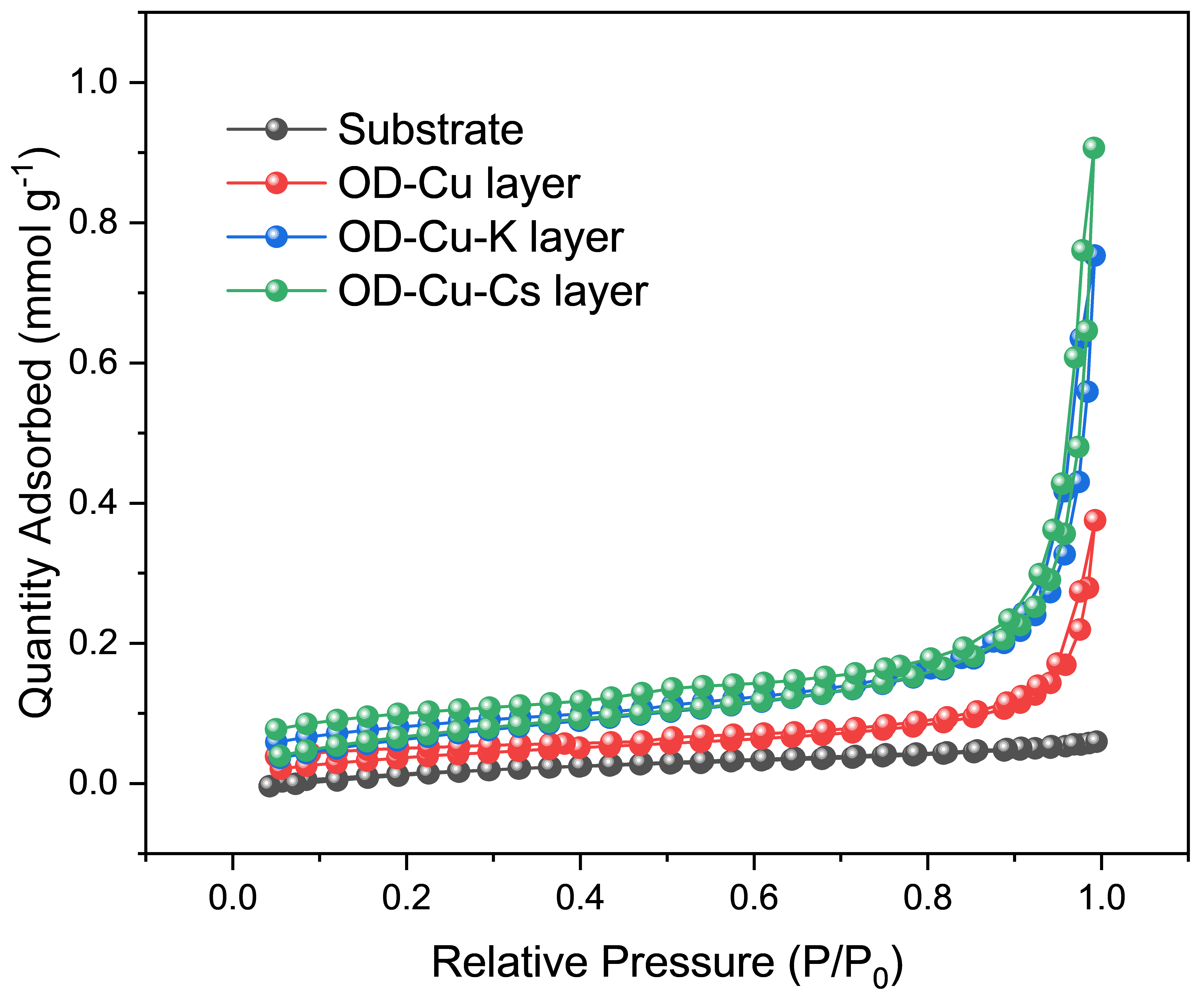


**Figure S4.** N_2_ adsorption and desorption isotherms of substrate, OD-Cu, OD-Cu-K, and OD-Cu-Cs layers.

200 nm

**
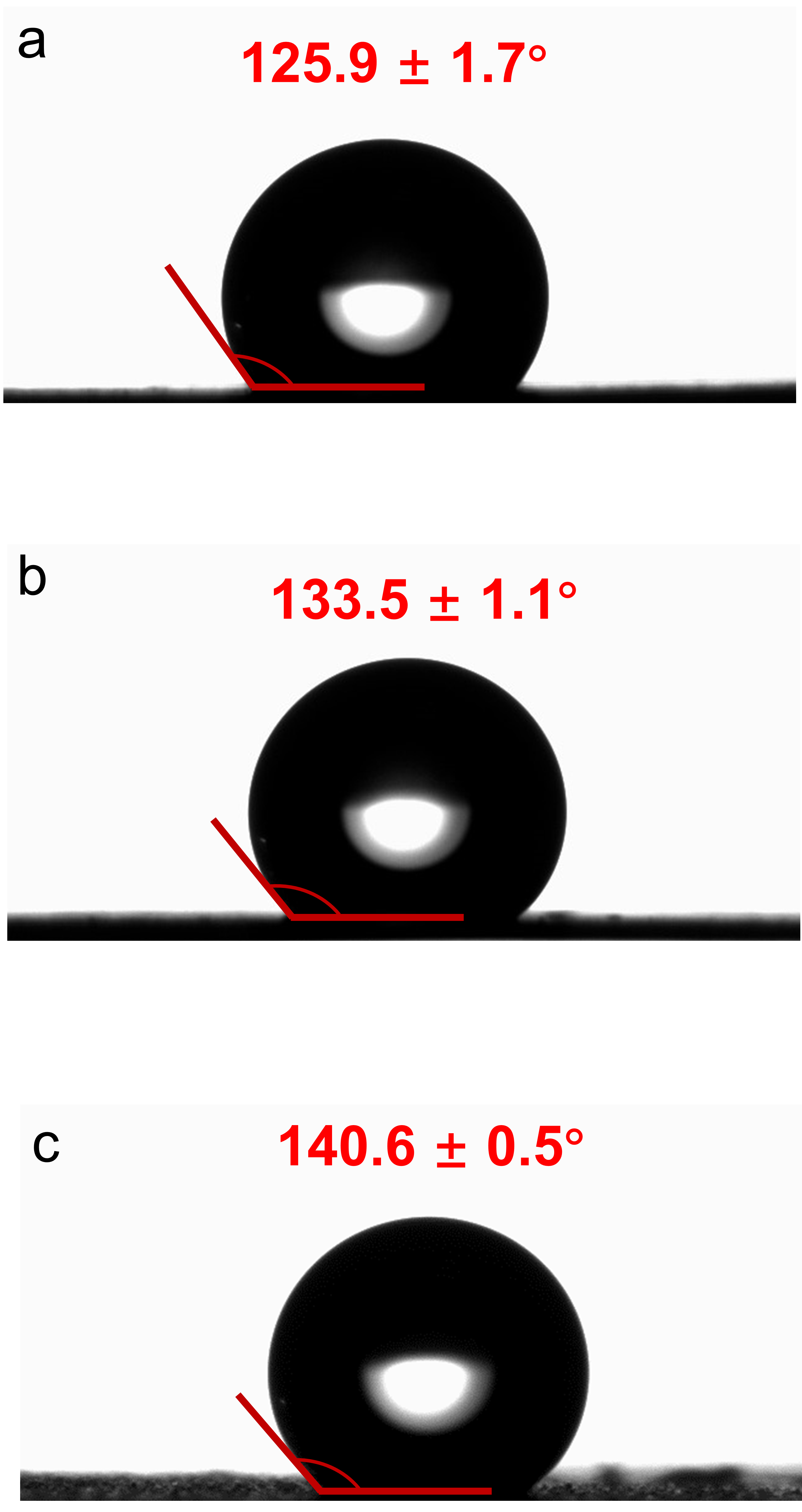
**

**Figure S5**. The contact angle of freshly-prepared (a) CuO, (b) CuO-K and (c) CuO-Cs layers.

**
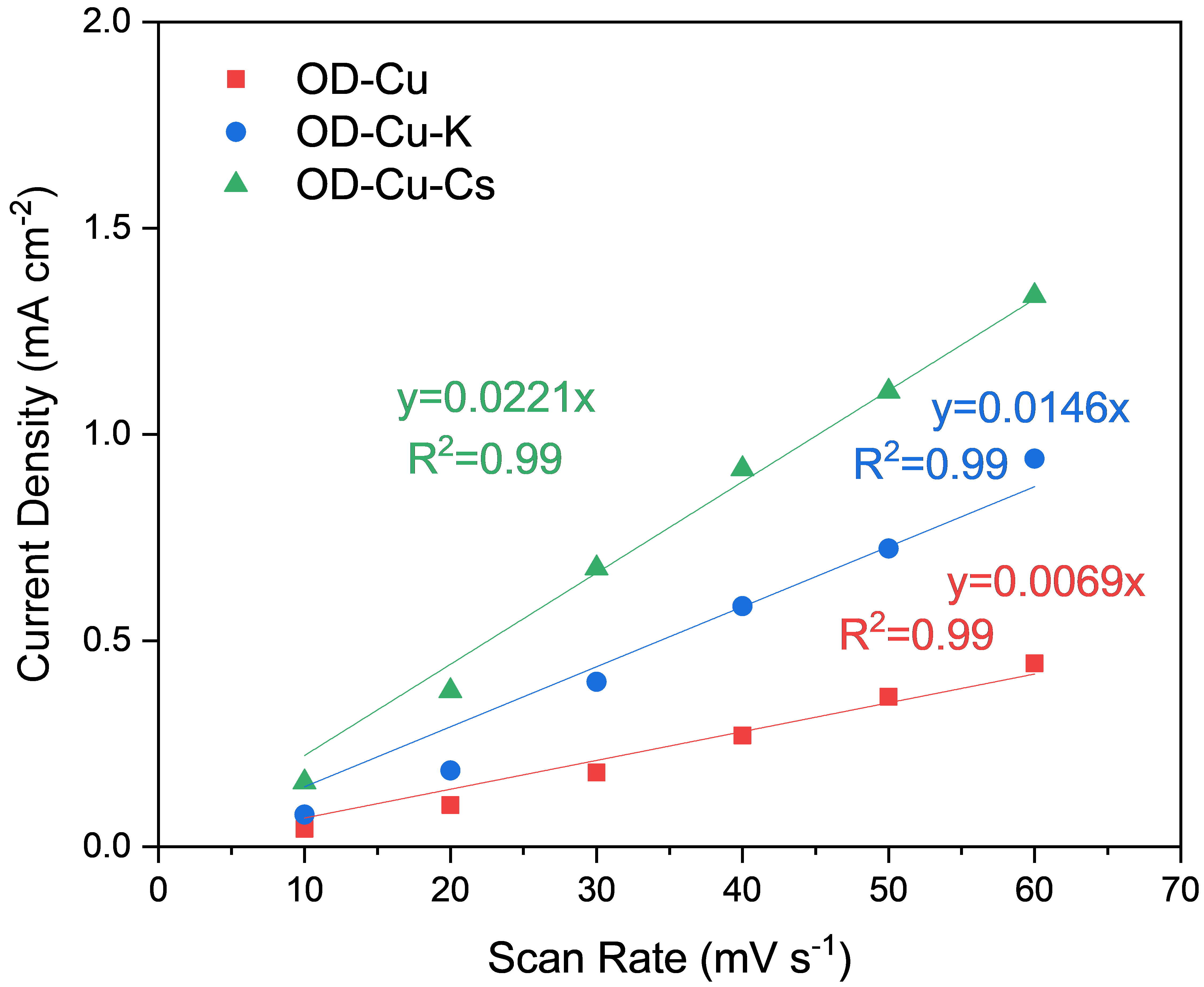
**

**Figure S6.** Determination of ECSAs of catalysts. The electrodes were pre-reduced at – 3 A cm^-2^ for 45 min before performing CV. Mass loadings of CuO, CuO-K and CuO-Cs were 2 mg·cm^−2^. Measurements were done in a flow cell electrolyzer using N_2_-saturated 1 M KOH. Double layer capacitive current density plotted against the scan rate for different electrodes. The slope of the linear regression line gives the double-layer capacitance.

**
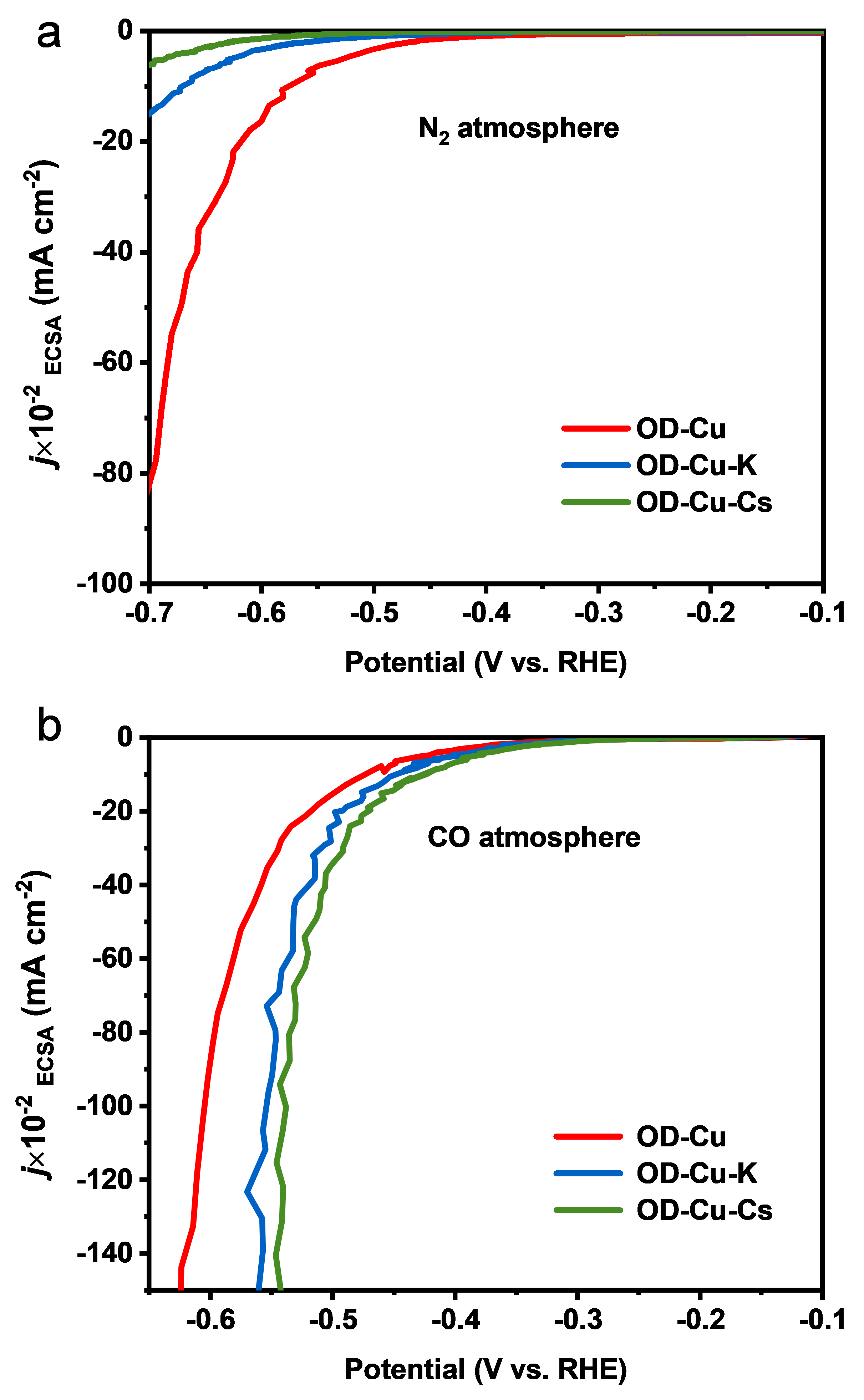
**

**Figure S7.** ECSA-normalized LSV curves of OD-Cu, OD-Cu-K, and OD-Cu-Cs in a) N_2_ and b) CO-saturated 1 M KOH. The current interrupt mode was used for IR-drop correction.

**~~
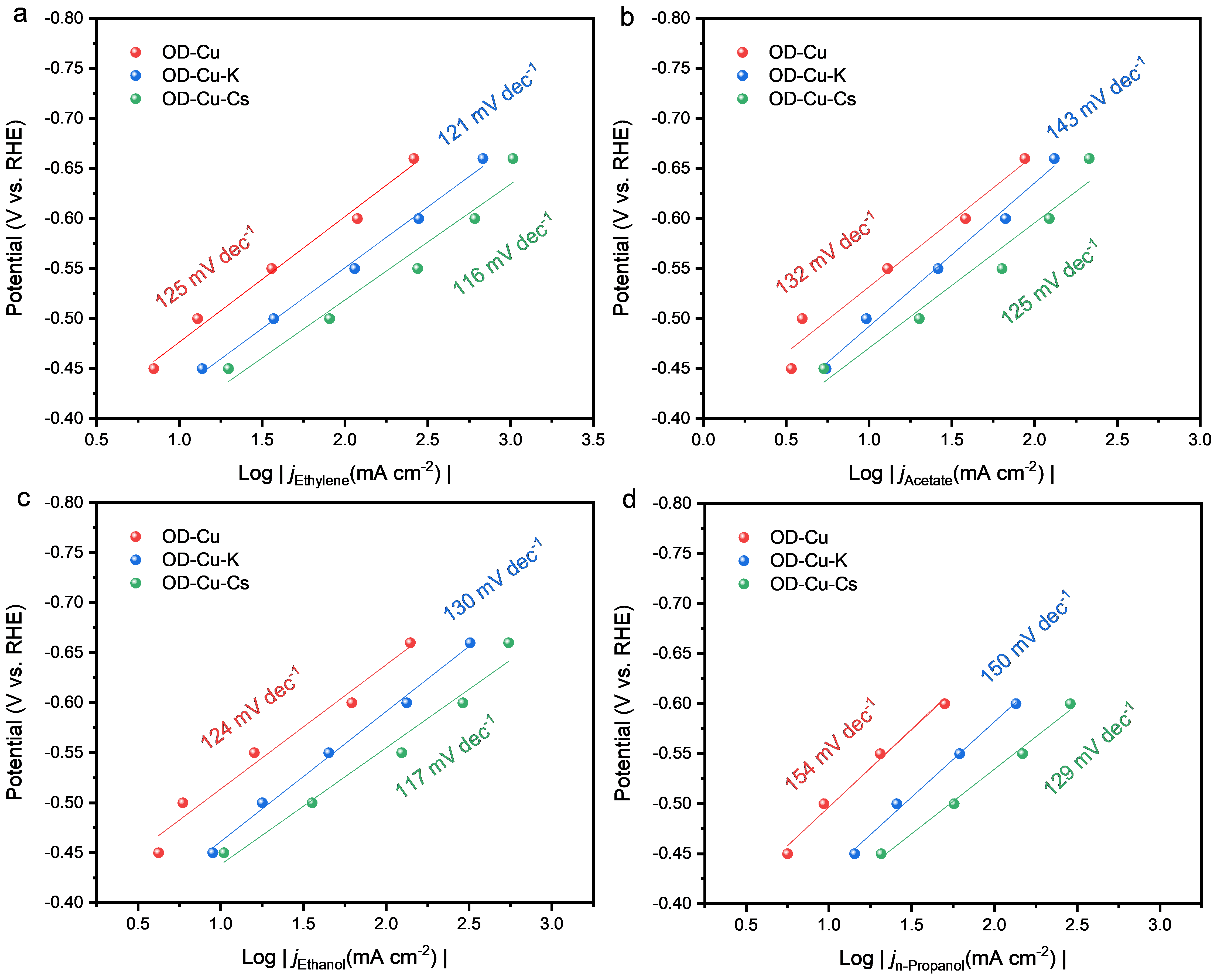
~~**

**Figure S8.** Tafel plots for the formation of a) ethylene, b) acetate, c) ethanol and d) *n*-propanol from CORR on OD-Cu, OD-Cu-K and OD-Cu-Cs.

**Reference**

[1] K. Phiwdang, S. Suphankij, W. Mekprasart, W. Pecharapa, *Energy Procedia* **2013**, *34*, 740-745.

[2] X. Wang, K. Klingan, M. Klingenhof, T. Möller, J. Ferreira de Araújo, I. Martens, A. Bagger, S. Jiang, J. Rossmeisl, H. Dau, P. Strasser, *Nat. Commun.* **2021**, *12*, 794.

[3] Y. Pang, J. Li, Z. Wang, C.-S. Tan, P.-L. Hsieh, T.-T. Zhuang, Z.-Q. Liang, C. Zou, X. Wang, P. De Luna, J. P. Edwards, Y. Xu, F. Li, C.-T. Dinh, M. Zhong, Y. Lou, D. Wu, L.-J. Chen, E. H. Sargent, D. Sinton, *Nat. Catal.* **2019**, *2*, 251-258.

[4] M. Sun, J. Cheng, M. Yamauchi, *Nat. Commun.* **2024**, *15*, 491.

[5] S. Zhao, O. Christensen, Z. Sun, H. Liang, A. Bagger, K. Torbensen, P. Nazari, J. V. Lauritsen, S. U. Pedersen, J. Rossmeisl, K. Daasbjerg, *Nat. Commun.* **2023**, *14*, 844.

[6] Y. Zhou, A. J. Martín, F. Dattila, S. Xi, N. López, J. Pérez-Ramírez, B. S. Yeo, *Nat. Catal.* **2022**, *5*, 545-554.

[7] C. W. Li, M. W. Kanan, *J. Am. Chem. Soc.* **2012**, *134*, 7231-7234.

[8] J. J. Lv, M. Jouny, W. Luc, W. Zhu, J. J. Zhu, F. Jiao, *Adv. Mater.* **2018**, *30*, 1803111.

[9] S. Kwon, J. Zhang, R. Ganganahalli, S. Verma, B. S. Yeo, *Angew. Chem. Int. Ed.* **2023**, *62*, e202217252.

[10] S. Hu, Y. Chen, Z. Zhang, S. Li, H. Liu, X. Kang, J. Liu, S. Ge, J. Wang, W. Lv, Z. Zeng, X. Zou, Q. Yu, B. Liu, *Small* **2024**, *20*, 2308226.

[11] Y. Cao, S. Chen, S. Bo, W. Fan, J. Li, C. Jia, Z. Zhou, Q. Liu, L. Zheng, F. Zhang, *Angew. Chem. Int. Ed.* **2023**, *62*, e202303048.

[12] M. Zheng, P. Wang, X. Zhi, K. Yang, Y. Jiao, J. Duan, Y. Zheng, S.-Z. Qiao, *J. Am. Chem. Soc.* **2022**, *144*, 14936-14944.

[13] C. Chen, X. Yan, Y. Wu, S. Liu, X. Zhang, X. Sun, Q. Zhu, H. Wu, B. Han, *Angew. Chem. Int. Ed.* **2022**, *61*, e202202607.

[14] F. P. García De Arquer, C.-T. Dinh, A. Ozden, J. Wicks, C. Mccallum, A. R. Kirmani, D.-H. Nam, C. Gabardo, A. Seifitokaldani, X. Wang, Y. C. Li, F. Li, J. Edwards, L. J. Richter, S. J. Thorpe, D. Sinton, E. H. Sargent, *Science* **2020**, *367*, 661-666.

[15] A. Inoue, T. Harada, S. Nakanishi, K. Kamiya, *EES Catal.* **2023**, *1*, 9-16.

[16] W. Ma, S. Xie, T. Liu, Q. Fan, J. Ye, F. Sun, Z. Jiang, Q. Zhang, J. Cheng, Y. Wang, *Nat. Catal.* **2020**, *3*, 478-487.

[17] C.-T. Dinh, T. Burdyny, M. G. Kibria, A. Seifitokaldani, C. M. Gabardo, F. P. García De Arquer, A. Kiani, J. P. Edwards, P. De Luna, O. S. Bushuyev, C. Zou, R. Quintero-Bermudez, Y. Pang, D. Sinton, E. H. Sargent, *Science* **2018**, *360*, 783-787.

[18] P. Wang, S. Meng, B. Zhang, M. He, P. Li, C. Yang, G. Li, Z. Li, *J. Am. Chem. Soc.* **2023**, *145*, 26133-26143.

[19] M. Ma, W. Deng, A. Xu, D. Hochfilzer, Y. Qiao, K. Chan, I. Chorkendorff, B. Seger, *Energy Environ. Sci.* **2022**, *15*, 2470-2478.

[20] M. P. Schellekens, S. J. Raaijman, M. T. M. Koper, P. J. Corbett, *Chem. Eng. J.* **2024**, *483*, 149105.
